# Fluctuating fire regimes and their historical effects on genetic variation in an endangered shrubland specialist

**DOI:** 10.1002/ece3.1811

**Published:** 2015-11-06

**Authors:** Hernán Vázquez‐Miranda, Kelly R. Barr, C. Craig Farquhar, Robert M. Zink

**Affiliations:** ^1^ Bell Museum and Department of Ecology, Evolution, and BehaviorUniversity of MinnesotaSt. PaulMinnesota55108USA; ^2^ Hopkins Marine StationStanford UniversityPacific GroveCalifornia93950USA; ^3^ Wildlife DivisionTexas Parks and Wildlife DepartmentAustinTexas78744USA

**Keywords:** Black‐capped vireo, conservation, fire ecology, savannas, statistical phylogeography

## Abstract

The Pleistocene was characterized by worldwide shifts in community compositions. Some of these shifts were a result of changes in fire regimes, which influenced the distribution of species belonging to fire‐dependent communities. We studied an endangered juniper–oak shrubland specialist, the black‐capped vireo (*Vireo atricapilla*). This species was locally extirpated in parts of Texas and Oklahoma by the end of the 1980s as a result of habitat change and loss, predation, brood parasitism, and anthropogenic fire suppression. We sequenced multiple nuclear loci and used coalescence methods to obtain a deeper understanding of historical population trends than that typically available from microsatellites or mtDNA. We compared our estimated population history, a long‐term history of the fire regime and ecological niche models representing the mid‐Holocene, last glacial maximum, and last interglacial. Our Bayesian skyline plots showed a pattern of historical population fluctuation that was consistent with changing fire regimes. Genetic data suggest that the species is genetically unstructured, and that the current population should be orders of magnitude larger than it is at present. We suggest that fire suppression and habitat loss are primary factors contributing to the recent decline of the BCVI, although the role of climate change since the last glacial maximum is unclear at present.

## Introduction

Ecological disturbances at many spatial and temporal scales can influence the distribution and genetic diversity of species and communities (Shapiro et al. 2004; Saab and Powell 2005; Campos et al. 2010; Banks et al. 2013). Fire is considered a major natural architect of the world's ecosystems and a driver of diversification. For example, Bytebier et al. (2011) found that a major shift in fire regime 20 million years ago led to a radiation of several clades of South African orchids. On a decadal timescale, fire suppression has been shown to influence the genetic structure of the eastern collared lizard (*Crotaphytus collaris*; Neuwald and Templeton 2013). Pereoglou et al. (2013) found that fire regime influenced genetic patterns at the landscape scale. Woodland savanna and shrubland biomes provide habitat for many organisms (Skarpe 1991). Savannas – a natural ecotone between grasslands and forest – depend on a critical balance of C_4_ grass accumulation and fire action purging dense understory and closed canopies that in turn promote the growth of grasses creating a positive feedback for savannas and fire (Beckage et al. 2009). In addition, shrublands more generally can be dependent on fire, especially in more mesic situations where sufficient moisture and soil types allow succession to woodlands that also over‐shadow a shrub understory. Despite these well‐known effects of fire in recent ecosystems, few studies have explored the long‐term relationship between organismal occurrence in shrublands and savannas and shifts in fire regime.

Fire‐dependent savanna habitats are one of the most “globally imperiled ecosystems” due to anthropogenic pressures that have reduced the historic 20 million ha of continuous distribution in the U.S.A. from Minnesota to Texas to 12,000 highly fragmented ha after European settlement (McPherson 1997, p. 34). One of those pressures was the suppression of fire. At a deeper timescale, North American savannas expanded after the last glacial maximum (ca. 21,000 ybp; LGM) when the fire cycles became more frequent in the Holocene around 10,000 ybp (Power et al. 2008; Marlon et al. 2009; Bryant 2012). Pollen records show that juniper–oak savannas in Texas also expanded after the LGM, becoming the dominant vegetation on Edwards' Plateau and contacting prairie grasslands from the American Midwest (Bryant 2012). These changes in the fire regime have been tied to events of rapid climate change between 15,000 and 10,000 ybp (Marlon et al. 2009) during the last glacial–interglacial transition in North America stemming from abrupt temperature changes, in particular the cooling at the Younger Dryas (~12,900–11,700 ybp, Steffensen et al. 2008). We expect changes in the amount of suitable habitat to affect long‐term population size and genetic diversity of species that occur in obligate fire‐dependent habitats. In particular, we expect population expansion to coincide with the advent of a more active and consistent fire regime for species that are intimately associated with fire‐dependent savannas and shrublands, followed by a very recent decline owing to anthropogenic factors, such as fire suppression and habitat loss.

To explore the impact of fire regime on population size, we investigated DNA sequence diversity in a vertebrate iconic of the juniper‐oak savannas of the southern Great Plains and more southerly thorn‐scrub, the BCVI (black‐capped vireo, *Vireo atricapilla*). BCVI is listed as Endangered under the U.S. Endangered Species Act. The species is a worthy proxy for assessing the historical effects of shifts in fire regimes because its preferred nesting habitat requires a balance of open grassland, brush patches, and short and solitary trees with little to no canopy cover (Grzybowski 1995), a habitat largely dependent on fire (Axelrod 1985, Beckage et al. 2009). There is a comparative framework of genetic markers for this species including allozymes (Fazio et al. 2004), microsatellites (Barr et al. 2008, 2011; Athrey et al. 2012a,b), and mitochondrial DNA (mtDNA, Zink et al. 2010). Although these markers provide resolution of relatively recent demographic events, little information exists on BCVI's deeper evolutionary history. Therefore, we sequenced multiple nuclear loci (Brito and Edwards 2009) and used climatic niche modeling to better understand BCVI population history over a longer time period than typically indexed by microsatellites and mtDNA. We determined whether genetic signatures of population fluctuations were consistent with major episodes in fire history, and we predicted that BCVI populations experienced at least one dramatic increase concomitant with the occurrence of increased fire frequency during the Holocene (10,000 ybp).

### Study species

**Figure 1:**
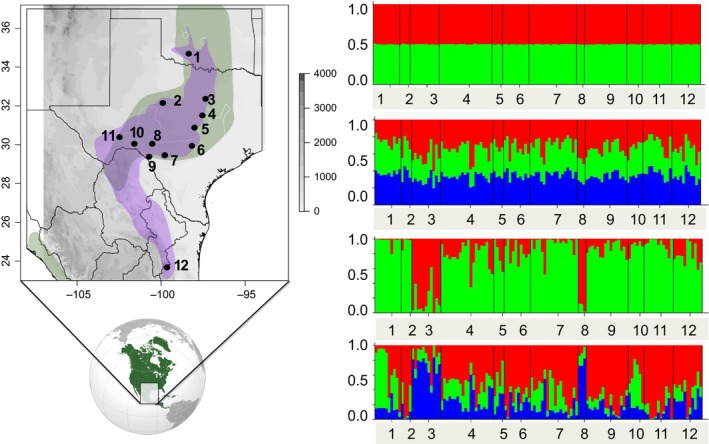
Breeding sampling localities in Oklahoma, Texas, Mexico, and Bayesian genotype cluster assignment of 124 individuals for *K *=* *2 and *K *=* *3 assuming admixture (top two) and no admixture (bottom two). Populations are arranged from north to south: Oklahoma (1), Camp Barkeley (2), Quail's Ridge (3), Fort Hood (4), Balcones Canyon Lands (5), San Antonio (6), Kerr Wildlife Management Area (7), Dobbs Mountain (8), Kickapoo Caverns (9), Devil's Ridge (10), Independence Creek (11), Mexico (12; it includes one individual from Coahuila, two from Nuevo Leon, and seven from Tamaulipas). The green polygon depicts current breeding (north) and wintering (south) distributions from NatureServe (http://www.natureserve.org). The purple polygon reflects newly discovered northern breeding populations that extend into northern Mexico, González‐Rojas et al. (2014). Legend in greyscale represents elevation in meters.

The BCVI is a monotypic species with a breeding distribution from Oklahoma (formerly as far north as Kansas) and Texas in the U.S.A. to Coahuila, Nuevo Leon, and Tamaulipas in Mexico (González‐Rojas et al. 2014), and they winter along the Pacific slope of western Mexico from Sonora to Oaxaca (Fig. 1; Grzybowski 1995; Howell and Webb 1995). They are listed as federally endangered in the United States (Ratzlaff 1987) and Mexico (SEMARNAT 2010), and vulnerable by the International Union for Conservation of Nature (IUCN SSC 2001). Only 191 breeding pairs were found in Oklahoma and Texas in 1987 (Wilkins et al. 2006), although by 2005 populations were estimated to include 6000 breeding males, owing in part to conservation efforts and more geographically comprehensive censuses that corrected the earlier inadequate census efforts (Wilkins et al. 2006; USFWS 2007). BCVI is considered a habitat specialist of juniper–oak savannas (Graber 1961) in central Texas and Oklahoma, and of arid thornscrub in SW Texas and NE Mexico (González‐Rojas et al. 2014), depending on dense, brushy vegetation for nesting. Thus, the species is affected by anthropogenic disturbance, including exotic ungulate grazers (Wilkins et al. 2006) and fire suppression. High tree density, resulting in closed canopy which in turn shades out shrubby understory, renders once‐productive breeding grounds unsuitable, especially in mesic vegetational communities of Texas and Oklahoma. Periodic fires reset the successional cycle in shrublands and savannas, providing or maintaining the dense interspersed thickets BCVIs prefer for nesting. Nonetheless, knowledge of the species' deeper environmental history is more fragmentary and we turned to insights gleaned from the distribution of genetic variation.

## Materials and Methods

### Samples and genetic procedures

We analyzed genetic samples from BCVI individuals used in previous studies that were collected from 12 localities (Barr et al. 2008; Zink et al. 2010; Table S1) representing the species' current distribution in Oklahoma, Texas, and Mexico (Fig. 1), which were augmented by DNA samples from recently collected tail feathers. We used previously extracted DNA or used a standard phenol chloroform protocol to improve DNA quality and quantity yield from tail feathers, followed by standard cold ethanol precipitation and final DNA elution in 40 *μ*L of TE buffer, stored at −20°C. In the tissue digestion phase, the DNA extraction buffer was supplemented with 40 *μ*L of DTT (dithiothreitol; 100 mg/mL) to break the disulfide bonds of keratinized feathers. We amplified and sequenced seven autosomal and two sex‐linked loci (Table 1). PCR (polymerase chain reaction) of each locus was accomplished in 12.5 *μ*L reactions of 3.25 *μ*L of water, 6.25 *μ*L of GoTaq Green Mastermix (Promega, Madison, WI), and 0.5 *μ*L of each primer at a concentration of 10 nmol/L, and 2 *μ*L of template DNA. All loci were amplified with the following touchdown thermocycler conditions: initial denaturation step of 95°C for 3 min, followed by five cycles of 95°C for 0.5 min, 58°C for 0.5 min and 72°C for 1 min, and repeating these previous steps changing the annealing temperature downward 2°C until it reached 52°C for 20 cycles and a final extension step of 72 ^o^ C for 6 min. PCR products were purified using a 3.4 *μ*L volume of the enzyme cocktail of Exonuclease I – Shrimp Alkaline Phosphatase method (ExoSAP, Affymetrix, Santa Clara, CA) following manufacturer instructions. PCR products of typical yield (100 ng/*μ*L) or higher as inspected by a visual comparison of the product band's brightness to a size DNA ladder of 100 base pairs (bps, Fisher Scientific, Fairlawn, NJ) in a 1% agarose gel were diluted by a factor of two after PCR purification to reach a concentration of 25 ng/*μ*L. For those amplicons with visibly low concentration, the dilution step was skipped and the product sequenced directly to avoid polymerase error mutations due to re‐amplification. Sequencing was carried out using the BigDye v3.1 termination protocol (Applied Biosystems, Foster City, CA) following recommendations by the manufacturer in an ABI 3730xl automated Sanger sequencer.

**Table 1:** Summary statistics per locus. Sex‐linked loci appear in bold. Significant values (*P *<* *0.05) are marked with an asterisk. Locus chromosome placement (Chr #), base pairs (# bps), allelic number (# alleles), allelic diversity (Hd), nucleotide diversity (*π*), and Fixation index (*F*_ST_)

Locus	Chr #	# bps	# alleles	Hd	*π*	*F*_ST_
**AdamS6**	**Z**	**291**	**5**	**0.3**	**0.001**	**0.025**
**IQGap2**	**Z**	**250**	**16**	**0.81**	**0.006**	**0.045**
Gapdh3	1	206	12	0.48	0.003	0.024
Lama2	3	275	7	0.51	0.002	0.022
TGFB2.5	3	236	15	0.82	0.006	0.038*
Fib5	4	242	12	0.67	0.005	0.04
Trop6	10	277	14	0.65	0.003	0.002
MC1R	11	293	13	0.72	0.005	0.054*
AETC	21	278	55	0.96	0.012	0.029*

Most DNA obtained from nonmolting feathers was fragmented and in low concentration, and amplification of products larger than 300 bps was unsuccessful. Therefore, we sequenced PCR products from blood using regular primers and redesigned specific primers for each locus flanking locus regions with polymorphic sites no longer than 300 bps (Table S2). Although for most loci excluded regions were adjacent to exons and invariable, for MC1R we chose a central range with the most variation. Amplification and sequencing for these smaller fragments were carried out as previously described.

### Allele phasing and recombination

DNA sequences were edited in SEQUENCHER 4.7 (Genecodes Inc., Ann Arbor, MI) and were verified to be of avian origin and the correct locus by comparing them to reference sequences in the NCBI GenBank database using BLAST searches. We also mapped loci to the chicken genome to verify their position in the avian genome using BLAST. For heterozygotes, we separated each allele using *in silico* phasing in the program PHASE 2.1 (Stephens et al. 2001) by interconverting the original aligned FASTA files in the program SEQPHASE (Flot 2010), running 100,000 generations with a burn‐in of 10,000. We tested for recombination between loci employing the Phi method (Bruen et al. 2006) in the program SPLITS TREE 4.3 (Huson and Bryant 2006).

### Testing for selection

Phylogenetic and phylogeographic inferences assume neutrality of the marker under survey, because selection can bias the nature and interpretation of polymorphisms detected. We tested for selection in a coalescent framework for all of our 10 loci using the HKA (Hudson–Kreitman–Aguadé) test (Hudson et al. 1987) with 10,000 simulations as implemented in the program HKA (J. Hey, https://bio.cst.temple.edu/~hey/software/software.htm#HKA), using a closely related species to BCVI, the slaty vireo (SLVI, *V. brevipennis*) as a reference for segregating sites and genetic distance (Slager et al. 2014).

### Estimating the age of BCVI

Understanding the evolutionary history of a species requires knowing how long it has been evolving independently as it diverged from its sister species. That is, it would be of little use to compare fire history and population genetic signatures if the genetic data actually represented the common ancestor of BCVI and its sister species. However, the phylogenetic relationships among all species of the family Vireonidae are unknown (Slager et al. 2014). Therefore, we reconstructed a phylogeny of representative lineages of vireos and close relatives to calculate divergence times with BEAST 1.7 (Drummond and Rambaut 2007) by concatenating our 10 loci using the ND2 gene for time calibration employing the avian rate of 1.3% substitutions per lineage, per million years (0.013 substitutions/lineage/million years, Arbogast et al. 2006) for 30 million generations and a burn‐in of 10%.

### Population genetic parameters and structure

To evaluate the demographic history of BCVI, it is useful to document current population structure. For example, if there were multiple evolutionarily units, each would have to be considered independently. Although a mtDNA (Zink et al. 2010) and microsatellite (Barr et al. 2008) found no structure, concern over the information content of loci (e.g., Galtier et al. 2009) led us to sample nuclear gene sequences. For the newly sequenced nuclear genes, we calculated measures of genetic variability using ARLEQUIN 3.2 (Excoffier et al. 2005): allele number, haplotype diversity (Hd), and nucleotide diversity (*π*). We calculated the fixation index (*F*_ST_) from allelic frequencies and its significance with 1000 bootstrap permutations in ARLEQUIN. We used the Bayesian method of individual genotype assignment in the program STRUCTURE 2.3 (Pritchard et al. 2000) to test the hypothesis of no deep structure (*K *=* *1) versus multiple clusters (*K *>* *1). We coded our nuclear loci as SNPs (single nucleotide polymorphisms; following Manthey et al. 2011) to calculate *K* with values between 1 and 20 clusters using the admixture (ancient panmixia) and nonadmixture models (ancient fragmentation) with sampling locality as a prior and both correlated and uncorrelated allele frequencies (similar results therefore only correlated frequencies reported), 20 repetitions per cluster with 1 × 10^6^ iterations and a burn‐in of 100,000 steps to ensure reproducibility (Gilbert et al. 2012), and a fixed lambda value (which was inferred by setting *K *=* *1 and allowing lambda to be estimated in an initial analysis). We used the Δ*K* method (Evanno et al. 2005) to identify the best estimate of *K* in STRUCTURE HARVESTER (Earl 2012). To evaluate IBD (isolation by distance), we plotted *F*_ST_ (1–*F*_ST_) versus the log of geographic distance among sites. We recognize this assumes demographic equilibrium, and we use the results as a heuristic indication of the relationship between population proximity and genetic distance.

### Historical demography

Most methods used to calculate population size changes are based on statistical deviation from a specific model (e.g., constant size, Rogers 1995) by permutations (Excoffier et al. 2005), and fluctuations of effective population size (*N*_*e*_) need to be assessed independently (Kuhner et al. 1998); however, it is now possible to estimate population sizes and fluctuations through time without a priori limitations from multiple loci (Ho and Shapiro 2011). We tested the population expansion hypothesis using EBSPs (Extended Bayesian Skyline Plots; Heled and Drummond 2008) implemented in BEAST 1.7 with three independent runs of 100 × 10^6^ generations and a burn‐in of 10 × 10^6^ steps. The model of evolution for each locus was calculated in the program JMODELTEST (Posada 2008). We used a coalescent genealogical model prior to calculate demographic growth and sizes, and for the time estimates, we scaled all the nuclear loci by their ploidy with relaxed clocks to the mtDNA dataset with a strict clock and the same substitution rate we used for the phylogeny. To scale nuclear loci among themselves, we used the avian rate of 0.00195 substitutions/lineage/million years for sex‐linked genes and 0.00184 for autosomal genes (Axelsson et al. 2004) with relaxed clocks with a standard deviation of 0.45. To transform *N*_*e*_ into absolute number of breeding individuals, we divided the population size estimate by generation time (Heled and Drummond 2008) assuming the age of reproduction of BCVI females to be 1 year (Grzybowski 1990). We constructed three different skyline plots to test for sex‐biased dispersal intrinsic of the locus: (i) “all evidence” included all 9 nuclear loci plus mtDNA from Zink et al. (2010), (ii) “male‐biased dispersal” including two sex‐linked loci alone, and (iii) “female‐biased dispersal” including only mtDNA.

To estimate fire history, we analyzed fluctuations of charcoal deposits in North America since the LGM. We plotted anomalous *Z*‐scores at charcoal sites from figure 5 in Power et al. (2008) for eastern and western North America onto our EBSP. An anomalous *Z*‐score is a standardized measurement of burned biomass that differs from present‐day charcoal; values of zero are similar; positive values indicate more burned biomass than present day; and negative values less burned biomass than present day. *Z*‐scores of ±2 are considered large charcoal anomalies (see Power et al. 2008 for details). Large positive *Z*‐scores should coincide with more frequent fires, and conversely, large negative values indicate historic fire reduction. There are records for only 35 LGM sites in the world (Power et al. 2008), and unfortunately, there are no standardized publically available *Z*‐scores for older time intervals; thus, we were unable to explore pre‐LGM correlations.

### Niche modeling

We estimated the potential climatic niche distribution of BCVIs through space and time to provide comparisons to results on population fluctuations suggested by genetic analyses. Our goal was not to determine which particular temperature or humidity variable(s) best explained distributions; instead, we were interested in relative distributional extents at different points in the past. We used locality records (*n* = 240) from the Breeding Bird Survey and used the maximum entropy method in MAXENT (Phillips et al. 2006). We used 19 bioclimatic layers from four time periods for breeding distributions: Present, mid‐Holocene (approximately 6000 years before present (ybp), LGM (21,000 ybp; Clark et al. 2009), and last interglacial (LIG, 120,000 ybp, Otto‐Bliesner et al. 2006) from the WorldClim database (“CCSM” worldclim.org; http://www.worldclim.org/paleo-climate). We clipped all layers to an area approximately 30% larger than the current breeding (−124 and −86 longitude, 42 and 5 latitude). Many of the climate layers are correlated (i.e., redundant), and there is concern over how to account for this (Shepard and Burbrink 2008; Brown and Knowles 2012). Hence, we ran MAXENT using several different combinations of variables. First, we ran MAXENT 10 times using default settings (except the number of iterations was increased to 1500 to allow the program to reach the default convergence threshold) and selected the climatic layers that accounted for 5% or more contribution to the model (BioClim variables: 1,2,9,10,12,16,17). We then reran the model using these layers and all locality records. Second, we chose the variables that offered the greatest permutation effects (2,9,11,16). Lastly, we computed correlations among the layers using ARCGIS10 and removed variables (5,8,9,15,17) that were highly correlated with others. We ran MAXENT five times for each set of variables and computed the average distributions (using the option cross‐validate). We used Diva‐GIS to display estimated distributions using a cutoff for presence at the “fixed cumulative value 5”. We made visual inspections of the estimated distributions from each of these three methods, and finding minimal differences we chose to illustrate distributions with the variables determined to be least correlated (correlation coefficients <0.8). We used ArcGIS to compute the area of predicted presence at the present time with a threshold occurrence of 0.15, at which level the present estimate of distribution is well predicted relative to the known distribution.

## Results

### Genetic variation and differentiation

We obtained sequences for nine nuclear loci for 124 individuals from 12 localities (Table 1; Fig. 1) with a range of 206–293 bps (after primer sites were trimmed), phased all alleles with a probability of at least 0.9, and found no evidence of recombination. Each locus was variable, exhibiting from 5 to 55 alleles. The HKA test was not significant (*X* ^*2*^ = 15.73, df = 18, *P *=* *0.61), corroborating selective neutrality of loci.

Haplotype diversity (Hd) varied from 0.96 (AETC locus) to 0.3 (ADAMS6). Nucleotide diversity (*π*) per locus followed a similar pattern (Table 1). Only three of the nine *F*_ST_ values were statistically significant; the overall average of 0.026 was not statistically significant.

In general, there is little geographic variation in levels of genetic variability (Table 2). For example, nucleotide diversity (*π*), which takes into account sample size differences, shows considerable uniformity, although the San Antonio sample is slightly more variable than the others.

**Table 2:** Summary statistics per locality (see Fig. 1 for locality information)

Locality	Ave. number of alleles surveyed	*π*	Hd	# alleles
1. OK	19.78	0.0043	0.68	5.2
2. CB	6.89	0.0044	0.57	3.1
3. QR	19.56	0.0036	0.59	4.8
4. FH	21.25	0.0039	0.52	5.1
5. BC	6.67	0.0043	0.69	3.3
6. SA	17.11	0.0064	0.71	5.9
7. KW	32.00	0.0051	0.68	7.6
8. DM	4.67	0.0032	0.44	2.1
9. KC	18.00	0.0046	0.59	5.1
10. DR	8.89	0.0042	0.68	3.6
11. IC	20.22	0.0050	0.68	5.8
12. MX	12.67	0.0054	0.74	4.5

**Figure 2:**
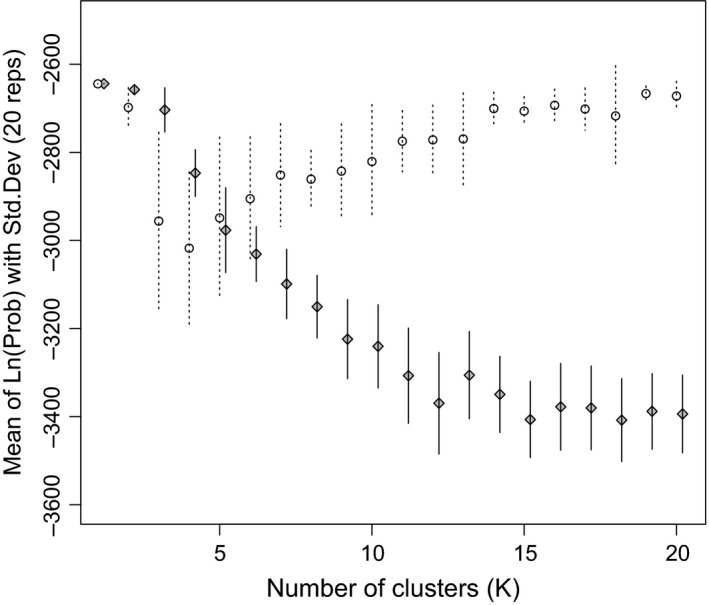
Likelihood values of inferred number of genetic clusters (*K*; 1–20). Plots are mean values of 20 repetitions per *K,* and vertical bars represent standard deviations: open circles and dotted lines represent population admixture assumption runs; filled diamonds and solid vertical bars represent no‐admixture assumption runs (with a jitter offset on *K* of 1.2 for visual purposes). The most likely value of *K* in every case is 1 regardless of prior information.

**Figure 3:**
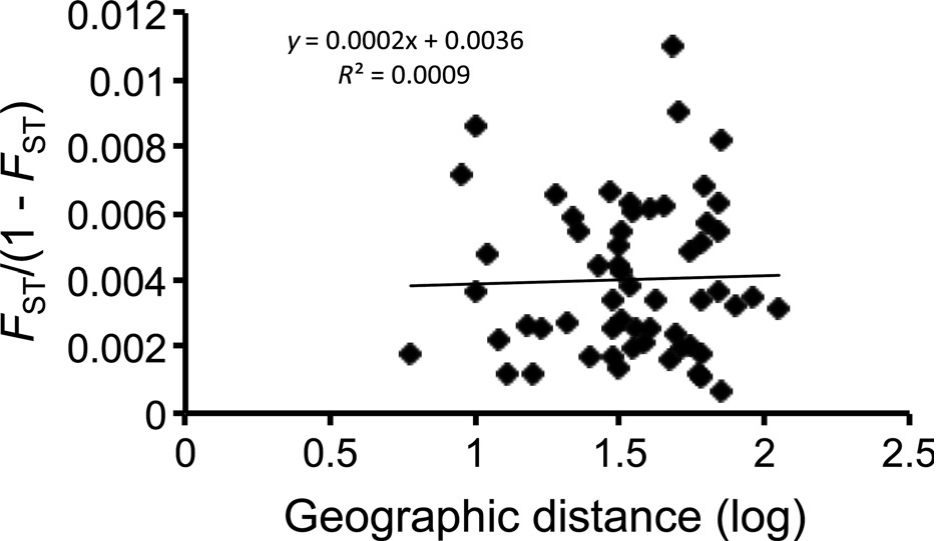
Isolation by distance pattern for 9 nuclear loci. The linear regression was not statistically significant (*P = *0.89).

The most likely number of genetic clusters is *K = *1. Δ*K* showed values of three or greater to be unlikely (Fig. 2). The Δ*K* statistic showed that the most likely *K* is 2 (results not shown); however, the Δ*K* statistic requires both lower and upper *K* likelihood values to be present, and therefore, *K *=* *1 and 2 cannot be assessed by this method because *K *=* *0 is nonsensical (Pritchard et al. 2000; Evanno et al. 2005). Nevertheless, *K *=* *2 is less likely than *K *=* *1 (Fig. 2) revealing no evidence of historical population structure. We found no evidence for IBD (*R*² = 0.0009, *P *=* *0.89; Fig. 3).

### Divergence of black‐capped vireo

Slager et al. (2014) confirmed the sister‐species relationship of BCVI and SLVI although a potentially related species, *V. nelsoni*, has yet to be included in a molecular analysis owing to a lack of available tissue samples. Our phylogenetic hypothesis (not shown) also suggest that BCVI and SLVI were sister species, and diverged during the Pliocene, ca. 4 million ybp (HPD 2.9–5.4 million ybp) suggesting a long independent history for BCVI and that the fire history influences the BCVI rather than its common ancestor with its sister taxon.

### Historical demography and fire history

**Figure 4:**
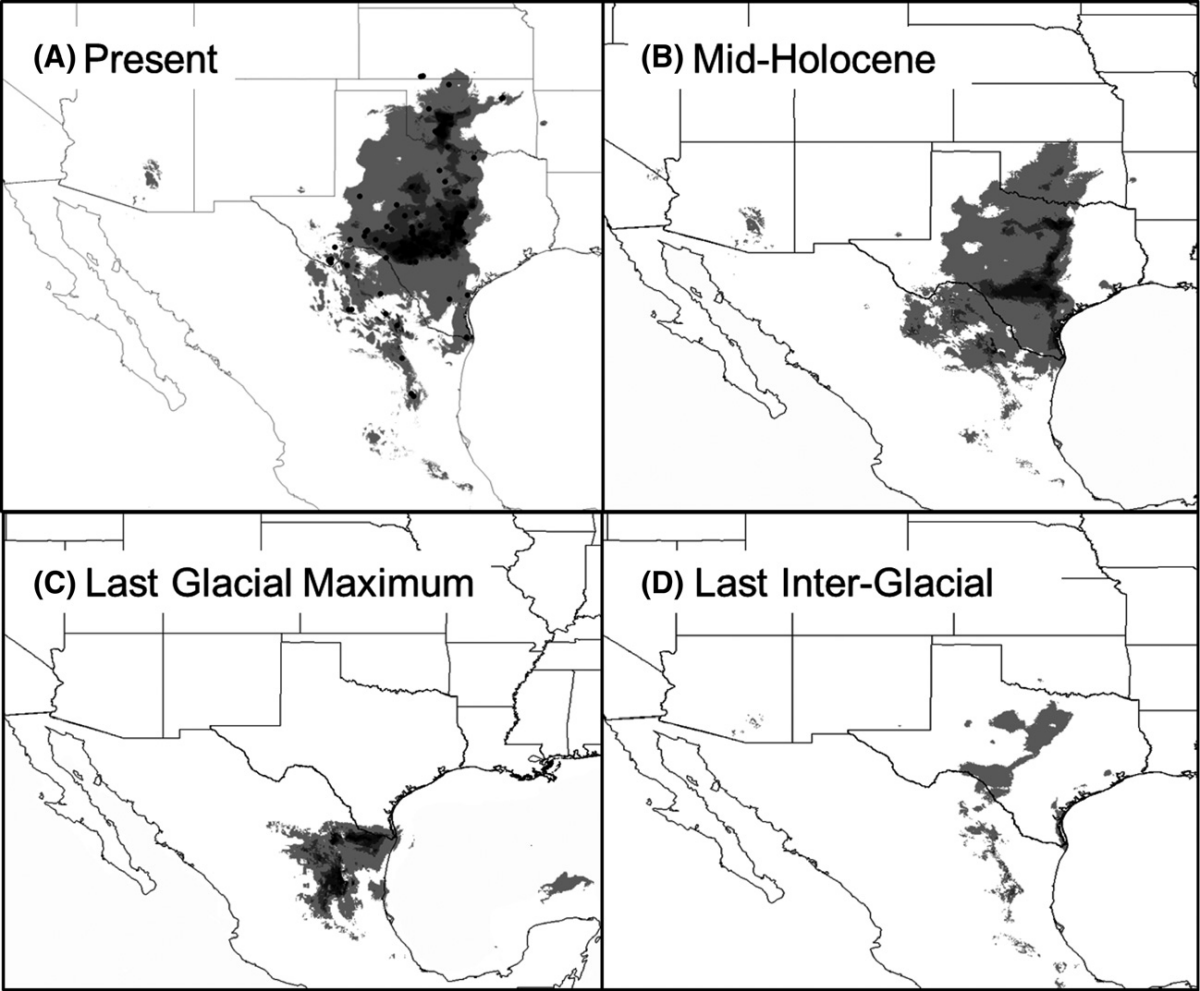
Predicted distributions based on climatic niche models derived from Maxent, showing shifting distributions over time. A–D panels represent four time periods used for niche modeling. Black areas are those for highest predicted occurrence, with decreasing amount of black of lesser predicted occurrence.

**Figure 5:**
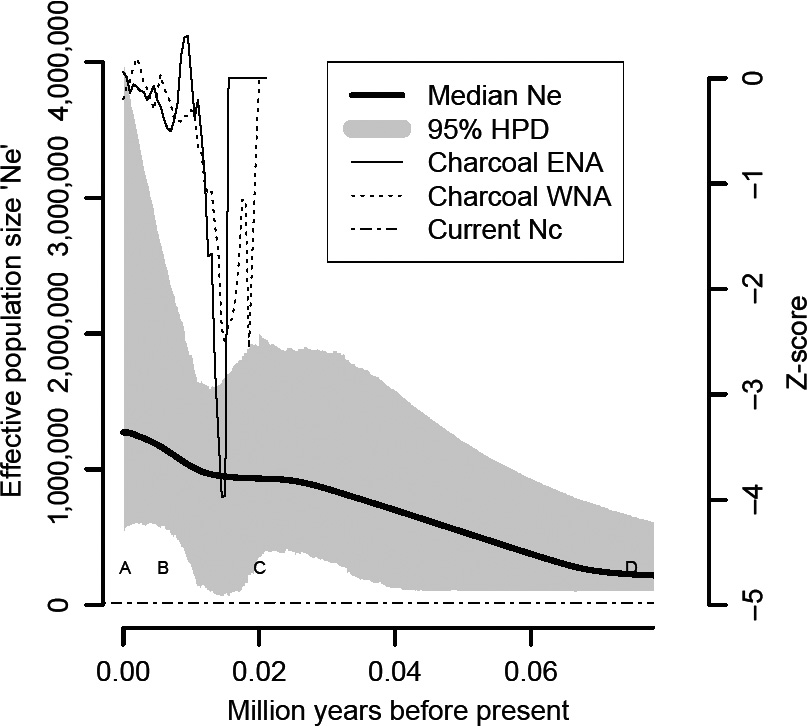
Extended Bayesian Skyline Plot; *Y*‐axis is effective population size (*N*_*e*_) in number of individuals, and *X*‐axis is in million years before present (ybp) with its 95% high posterior density (HPD). The anomalous *Z*‐score fluctuations represent differences in burned biomass (charcoal) to present‐day charcoal deposits since the LGM (fig. 5 in Power et al. 2008) for western (WNA) and eastern (ENA) North America. The dotted‐dashed line represents the current census size (*N*_*c*_; Wilkins et al. 2006). Only the last 70,000 years are shown. A = Present, B = mid‐Holocene, C = LGM, and D = LIG (see Fig. 4).

Using all nuclear loci and mtDNA, we found strong evidence for two effective population size change events (median = 1.8, 1–3; 95% high posterior density [HPD]; Fig. 5). The first increase after the Lower Pleniglacial (70,000 ybp) with a posterior mean estimate of *N*_*e*_ = 249,580 breeding individuals (100,000–742,000; 95% HPD). We note that it is the entire 95% HPD that yields this inference, not the median line. Before the LGM (35,000 ybp) *N*_*e*_ was 780,000 individuals (181,000–1,780,000; 95% HPD) and remained constant until glacial retreat. At the end of the LGM, we found a decrease in population size during the last glacial–interglacial transition with the lowest HPD of 38,000 individuals at ~14,000 ybp. The second increase happened at the beginning of the Holocene (10,000 ybp) with *N*_*e*_ **= 997,000 individuals (94,000–1,660,000; 95% HPD). Present‐day *N*_*e*_ was = 1,270,000 individuals (508,000–4,191,000 95% HPD). The most recent common ancestor (MRCA) of all sampled alleles can be traced back to the Early Pleistocene, 1.74 million ybp (median.root.height, 900,000–2,480,000 95% HPD), and BCVI populations were constant without significant fluctuations before 70,000 ybp, although it is possible that a lack of sufficient mutations in this period limits inferences (Fig. 4).

Historical charcoal‐record fluctuations coincide with the episodes of population increase and decrease through time as previously described. In North America, charcoal deposits during the LGM were similar to present day and reduced significantly (anomalous Z‐scores of −2 and −4 in eastern and western North America, respectively; fig. 5 in Power et al. 2008), concomitant with the climatic fluctuations after the LGM (Steffensen et al. 2008; Marlon et al. 2009). The charcoal deposits increased between 12,000 and 9000 ybp after the cooling of Younger Dryas period (Fig. 5; see also fig. 5 in Power et al. 2008).

Demographic trends were maintained even after removing the autosomal loci (Fig. S1). Nevertheless, use of mtDNA or sex‐linked loci alone were unable to reject the constant size population hypothesis due to their large posterior credibility intervals encompassing zero (Fig. S1), showing that potentially sex‐biased dispersal markers are not driving the analysis.

### Niche models

Niche models predicted a restricted breeding distribution at the LIG relative to today, an increase in suitable habitat at the LGM in northern Mexico, and a distribution at the mid‐Holocene that resembles the present distribution of breeding habitat in Texas and Oklahoma (ca. 30,000,000 ha) that altogether correspond to a post‐Pleistocene expansion of grasslands and juniper–oak savannas (Hofreiter and Stewart 2009; Bryant 2012). For all models, the area under curve AIC values exceeded 0.94, suggesting that the models performed satisfactorily.

## Discussion

### Distribution and historical demography through space and time

Our goal was to reconstruct a long‐term picture of the species' history using inferences from variation among nuclear DNA sequences. Knowing that a species has undergone historical fluctuations in size for natural reasons can lend insight into more recent population trends. We were interested in assessing the relationship between fire history and population trends in the BCVI, a species for which the extent of preferred habitat is at least partly fire dependent.

Our spatial distribution models provide support for a long‐term response of vireo populations to fire history (Fig. 5). Our distribution predictions through time showed relatively little breeding habitat at the LIG followed by an increase at the LGM in northern Mexico. This increase in habitat availability after the LGM and its expansion northwards into Texas, Oklahoma, and Kansas into the Holocene and present day is congruent with northwards expansion of temperate species during warmer times (Hofreiter and Stewart 2009) and an increase of pollen records associated with plant species characteristic of juniper‐oak savannas of the Edwards Plateau after 10,000 ybp (Bryant 2012). Given that the LGM, mid‐Holocene and Present models are snapshots of three points in time, we cannot detect a precise moment when an increase in population size occurred although it appears to have been between the end of the LGM and the mid‐Holocene. Our EBSP analyses suggest that the timing of two population expansions was 60,000 and 10,000 ybp, corresponding, respectively, to warmer climates after the Lower Pleniglacial and beginning of the Holocene. These results support the hypothesis that a shifting fire regime before and after glacial periods resulted in increasing savanna and shrubland habitat conducive to BCVI. In addition, Bayesian analyses require large numbers of alleles to detect historical demographic changes (Heled and Drummond 2008). Thus, the wide credibility intervals in our EBSPs based on one and two loci are an artifact of reducing allele (sample) size and highlight the importance of including multiple loci in phylogeographic studies to document population fluctuations.

Fire can influence groups with varying dispersal abilities in different ways. Arthropod and small mammal species likely perish during major fires whereas birds likely avoid fire by flying to different areas (Whelan 1995). We found that concomitant fluctuations between fire and population size illustrate the potential of fire to influence historical population structure of even highly vagile species such as the BCVI. Our findings suggest that this relationship is an old one, but that the relationship persists. BCVIs were found recently in areas considered unsuitable habitat in northern Texas after fires consumed large parts of the woodlands (H. Mathewson Pers. Comm.).

Given the spotty distribution of BCVI (the reason for its listing as Endangered) relative to our estimated current distribution, it would seem that human alteration of habitat has contributed significantly to the recent declines of the BCVI (Barr et al. 2008; Athrey et al. 2012a). Our molecular assessment of BCVI history provides a perspective that might otherwise be lacking for assessing current population trends. The question is what is the magnitude of recent population declines? Assuming the average ratio of *N*_*e*_ to census population sizes (*N*_*c*_) of 0.1 reported in empirical wildlife studies (Frankham 1995; Palstra and Ruzzante 2008), our results suggest the potential existence of 12,000,000 BCVIs, 1000 times more than currently estimated (Wilkins et al. 2006). Our estimates of the extent of predicted distribution at the present and the mid‐Holocene, ca. 3 × 10^7^ ha, yield a potential population size of 20,000,000–8,300,000 individuals, assuming a territory size of 1.5–3.6 ha/pair, respectively (Graber 1961; Tazik 1991). However, few species with a total population size of this magnitude (Partner's in Flight, http://rmbo.org/pifpopestimates/Database.aspx, accessed 6 July 2015) currently have such a limited geographic distribution. Nonetheless, our results give perspective to potential differences between current and historical population sizes. It seems likely that without human alteration of their habitat, BCVIs should be much more common at present, although not necessarily a great deal more widespread (Fig. 4). Thus, the estimated reduction and fragmentation of North American shrublands to 6% of their original extent seems to have had a significant impact on modern‐day populations of BCVIs. As a caveat, we note that if we assume a generation time of 2 years, there are still many fewer birds at present than were estimated to exist at the maximum extent of fire‐induced habitat.

In addition to fire suppression, brood parasitism and predation (Smith et al. 2012) have been implicated in reduction of current BCVI populations. Brown‐headed cowbirds are a native species and before the 19th century were found predominantly in the prairies west of the Mississippi River; in 100 years they spread throughout eastern North America (Brittingham and Temple 1983). In addition, cowbirds are present in Pleistocene fossil deposits from California to Florida (Miller 1947; Oswald and Steadman 2011). However, the eastward spread of brown‐headed cowbirds into BCVI breeding areas has had a recent negative effect on some populations (Wilsey et al. 2014), although the overall magnitude of the effect is unclear given that cowbirds have co‐occurred with vireos at least since the LGM. In addition, there is evidence of a consistent predatory species pool of mammals, reptiles, and raptors since the LGM and in Holocene localities in Texas (Blair 1952; Smith et al. 2012) and the American Southwest (Oswald and Steadman 2011); thus, the historical appearance of a new major predator seems unlikely.

### Roles for molecular markers

Although it was not our principal goal, given recent debates (Zink and Barrowclough 2008; Edwards and Bensch 2009; Galtier et al. 2009) over the efficacy of various molecular markers, we briefly compare results from the molecular markers surveyed to date, excluding allozymes (which are well known to lack resolving power at the population level in birds).

We found similar results for all molecular markers, including microsatellite allele frequencies (Barr et al. 2008; Athrey et al. 2012a), mtDNA sequences (Zink et al. 2010), and nuclear gene sequences (this study). We found no signatures of historical population structure in any of the loci we explored. The average *F*_ST_ of 0.026 value that we found was consistent with that observed in microsatellites (Barr et al. 2008) and mtDNA (Zink et al. 2010). The individual genotype assignment test rejected the population structure hypothesis as well (*K *=* *1). Hence, although there might be recent local restrictions to gene flow (Barr et al. 2008; Athrey et al. 2012a,b), there are not multiple evolutionarily significant units within BCVI despite existing as a single distinct lineage for over 1 million ybp. The geographic area occupied as at least the LIG is likely too small for isolating barriers to cause significant historical structuring. The plots of IBD for microsatellites (Barr et al. 2008), mitochondrial DNA (Zink et al. 2010), and nuclear gene sequences (Fig. 3) are all consistent with at most a slight if any effect of distance. Thus, results from analyses of mtDNA, microsatellites, and nuclear gene sequences were consistent.

## Data accessibility

DNA sequences are deposited in GenBank. See Supporting Information.

Locality data: http://datadryad.org/review?doi=doi:10.5061/dryad.j6ft6

## Conflict of Interest

None declared.

## Supporting information


**Table S1.** Sample information, localities, and Genbank accession numbers
**Table S2.** Primers used in loci amplification
**Figure S1.** Comparisons of Extended Bayesian Skyline Plot demographies.
