# Supplementary material for: Fluctuating fire regimes and their historical effects on genetic variation in an endangered shrubland specialist
Source: Ecol Evol. 2015 Nov 6;5(23):5487–98. doi: 10.1002/ece3.1811 (PMC4813106; doi:10.1002/ece3.1811)
Supplement: Supplementary file 1 — Table S1. Sample information, localities, and Genbank accession numbers Table S2. Primers used in loci amplification Figure S1. Comparisons of Extended Bayesian Skyline Plot demographies. [file ECE3-5-5487-s001.doc]

Supplementary Materials

**Fluctuating fire regimes and their historical effects on genetic variation in an endangered savanna specialist**

Hernán Vázquez-Miranda, Kelly R. Barr, C. Craig Farquhar, and Robert M. Zink

Supplementary Table 1. Sample information, localities, and Genbank accession numbers

Supplementary Table 2. Primers used in loci amplification

Supplementary Figure 1. Comparisons of Extended Bayesian Skyline Plot demographies

**Supplementary Table 1**. **Sample, locality, and locus information with Genbank accession numbers**. Number preciding a state name correspond to locality numbers (Fig.1 in main text). Data and sample source codes are Barr *et al.* 2008 (1), Zink *et al.* 2010 (2), and new for this study (3). Ten loci sampled: ND2 is NADH deshydrogenase subunit 2 from mitochondrial DNA; MC1R is Melanocortin receptor 1; TGFB2-I5 is Transforming growth factor β-chain 2 – intron five; FIB5 is Beta fibrinogen - intron 5; IQGAP2 is Ras GTPase-activating-like protein; ADAMTS6 is ADAMTS6 variant 2; AETC is τ-crystalline/alpha enolase – intron 8; GAPDH is Glyceraldehyde-3-phosphate dehydrogenase – intron 11; LAMA2 is Lam2 Lamin gene – intron 3; and TROP6 is Tropomyosin – intron 6. Nuclear genes: IQGAP2 and ADAMTS6 are Z-linked loci, the rest are autosomal. Dashed lines (-) indicate samples did not amplify for a particular locus.

| SAMPLE | Species | Country | State | Locality | Source | ND2 | MC1R | TGFB2-I5 | FIB5 | IQGAP2 | ADAMT56 | AETC | GADPH | LAMA2 | TROP6 |
| --- | --- | --- | --- | --- | --- | --- | --- | --- | --- | --- | --- | --- | --- | --- | --- |
| VA001 | Vireo atricapilla | USA | 01. Oklahoma | Fort Sill | 2 | FJ560600 | KT883303 | KT883435 | KT882864 | KT883015 | KT882634 | KT882715 | KT882887 | KT883282 | KT883528 |
| VA002 | Vireo atricapilla | USA | 01. Oklahoma | Fort Sill | 2 | FJ560601 | KT883298 | KT883430 | KT882806 | KT883028 | KT882660 | KT882777 | KT882921 | KT883184 | KT883506 |
| VA003 | Vireo atricapilla | USA | 01. Oklahoma | Fort Sill | 2 | FJ560602 | KT883299 | KT883429 | KT882803 | KT883032 | KT882659 | KT882776 | KT882923 | KT883280 | KT883512 |
| VA004 | Vireo atricapilla | USA | 01. Oklahoma | Fort Sill | 2 | FJ560603 | KT883304 | KT883431 | KT882854 | KT882990 | KT882640 | KT882717 | KT882885 | KT883182 | KT883444 |
| VA005 | Vireo atricapilla | USA | 01. Oklahoma | Fort Sill | 2 | FJ560604 | KT883305 | KT883421 | KT882804 | KT882989 | KT882700 | KT882719 | KT882884 | KT883241 | KT883508 |
| VA006 | Vireo atricapilla | USA | 01. Oklahoma | Fort Sill | 2 | FJ560605 | KT883340 | KT883420 | KT882805 | - | KT882693 | KT882716 | KT882957 | KT883183 | KT883536 |
| VA007 | Vireo atricapilla | USA | 01. Oklahoma | Fort Sill | 2 | FJ560606 | KT883359 | KT883372 | KT882834 | KT883033 | KT882638 | KT882733 | KT882922 | KT883293 | KT883510 |
| VA008 | Vireo atricapilla | USA | 01. Oklahoma | Fort Sill | 2 | FJ560607 | KT883296 | KT883434 | KT882821 | KT883031 | KT882699 | KT882747 | KT882975 | KT883186 | KT883509 |
| VA009 | Vireo atricapilla | USA | 01. Oklahoma | Fort Sill | 2 | FJ560608 | KT883336 | KT883371 | KT882853 | KT883059 | KT882689 | KT882718 | KT882879 | KT883208 | KT883467 |
| VA010 | Vireo atricapilla | USA | 01. Oklahoma | Fort Sill | 2 | FJ560609 | KT883339 | KT883404 | KT882867 | KT882985 | KT882697 | KT882729 | KT882963 | KT883214 | KT883468 |
| VA058 | Vireo atricapilla | USA | 02. Texas | Camp Barkeley | 2 | FJ560654 | KT883328 | KT883373 | KT882822 | KT883012 | - | - | KT882876 | KT883207 | KT883448 |
| VA059 | Vireo atricapilla | USA | 02. Texas | Camp Barkeley | 2 | - | - | - | KT882836 | KT883023 | KT882712 | - | KT882933 | KT883271 | KT883498 |
| VA060 | Vireo atricapilla | USA | 02. Texas | Camp Barkeley | 2 | FJ560655 | KT883350 | KT883407 | KT882820 | KT882993 | KT882706 | KT882730 | KT882917 | KT883273 | KT883449 |
| VA061 | Vireo atricapilla | USA | 02. Texas | Camp Barkeley | 2 | FJ560656 | KT883365 | KT883388 | KT882824 | KT883050 | KT882702 | KT882726 | KT882935 | KT883269 | KT883537 |
| VA125 | Vireo atricapilla | USA | 03. Texas | Quail's Ridge | 2 | FJ560695 | - | - | KT882866 | KT883074 | - | KT882778 | KT882956 | KT883230 | KT883483 |
| VA126 | Vireo atricapilla | USA | 03. Texas | Quail's Ridge | 2 | FJ560696 | KT883325 | KT883376 | KT882798 | KT883037 | KT882646 | KT882734 | KT882954 | KT883212 | KT883518 |
| VA127 | Vireo atricapilla | USA | 03. Texas | Quail's Ridge | 2 | FJ560697 | KT883368 | KT883402 | KT882851 | KT883000 | KT882709 | KT882752 | KT882964 | KT883266 | KT883488 |
| VA128 | Vireo atricapilla | USA | 03. Texas | Quail's Ridge | 2 | FJ560698 | KT883348 | KT883380 | - | KT883064 | KT882655 | KT882787 | KT882913 | KT883267 | KT883445 |
| VA129 | Vireo atricapilla | USA | 03. Texas | Quail's Ridge | 2 | FJ560699 | KT883353 | - | - | KT883002 | KT882674 | KT882789 | KT882942 | KT883268 | KT883443 |
| VA130 | Vireo atricapilla | USA | 03. Texas | Quail's Ridge | 2 | FJ560700 | KT883324 | KT883406 | - | KT883025 | KT882652 | KT882758 | KT882893 | KT883213 | KT883520 |
| VA131 | Vireo atricapilla | USA | 03. Texas | Quail's Ridge | 2 | FJ560701 | KT883367 | KT883377 | KT882865 | KT882991 | KT882654 | - | KT882906 | KT883279 | KT883526 |
| VA132 | Vireo atricapilla | USA | 03. Texas | Quail's Ridge | 2 | FJ560702 | KT883322 | KT883432 | KT882800 | KT883038 | KT882653 | KT882735 | KT882953 | KT883284 | KT883505 |
| VA133 | Vireo atricapilla | USA | 03. Texas | Quail's Ridge | 2 | FJ560703 | KT883366 | KT883383 | KT882830 | KT883003 | KT882708 | KT882779 | KT882910 | KT883215 | KT883439 |
| VA134 | Vireo atricapilla | USA | 03. Texas | Quail's Ridge | 2 | FJ560704 | - | KT883437 | KT882848 | KT883063 | KT882651 | - | KT882894 | KT883283 | KT883487 |
| VA135 | Vireo atricapilla | USA | 03. Texas | Quail's Ridge | 2 | FJ560705 | - | KT883389 | KT882849 | KT883001 | KT882647 | - | KT882911 | KT883246 | KT883513 |
| VA023 | Vireo atricapilla | USA | 04. Texas | Fort Hood | 2 | FJ560620 | - | - | - | - | - | - | - | KT883178 | - |
| VA024 | Vireo atricapilla | USA | 04. Texas | Fort Hood | 2 | FJ560621 | - | - | - | - | - | - | - | KT883290 | - |
| VA030 | Vireo atricapilla | USA | 04. Texas | Fort Hood | 2 | FJ560627 | - | - | - | - | - | - | - | KT883201 | - |
| VA035 | Vireo atricapilla | USA | 04. Texas | Fort Hood | 2 | FJ560632 | - | - | - | - | - | - | - | KT883237 | KT883457 |
| VA036 | Vireo atricapilla | USA | 04. Texas | Fort Hood | 2 | FJ560633 | - | - | - | - | - | - | - | KT883270 | - |
| VA037 | Vireo atricapilla | USA | 04. Texas | Fort Hood | 2 | FJ560634 | - | - | - | - | - | - | - | KT883272 | - |
| VA039 | Vireo atricapilla | USA | 04. Texas | Fort Hood | 2 | FJ560636 | - | - | - | - | - | - | - | KT883232 | KT883458 |
| VA040 | Vireo atricapilla | USA | 04. Texas | Fort Hood | 2 | FJ560637 | - | - | - | - | - | - | - | KT883238 | KT883531 |
| VA041 | Vireo atricapilla | USA | 04. Texas | Fort Hood | 2 | FJ560638 | - | - | - | - | - | - | - | KT883206 | KT883541 |
| VA042 | Vireo atricapilla | USA | 04. Texas | Fort Hood | 2 | FJ560639 | - | - | - | - | - | - | - | KT883180 | KT883469 |
| VA136 | Vireo atricapilla | USA | 04. Texas | Fort Hood | 3 | KT883546 | KT883320 | - | KT882831 | KT883014 | KT882692 | KT882746 | KT882925 | KT883179 | KT883540 |
| VA137 | Vireo atricapilla | USA | 04. Texas | Fort Hood | 3 | KT883547 | KT883358 | - | KT882833 | - | KT882687 | KT882741 | KT882889 | KT883185 | KT883517 |
| VA138 | Vireo atricapilla | USA | 04. Texas | Fort Hood | 3 | KT883548 | KT883345 | - | KT882832 | KT883048 | KT882683 | KT882731 | KT882924 | KT883262 | KT883491 |
| VA139 | Vireo atricapilla | USA | 04. Texas | Fort Hood | 3 | KT883549 | KT883356 | - | KT882826 | KT883040 | KT882701 | KT882784 | KT882918 | KT883261 | KT883440 |
| VA140 | Vireo atricapilla | USA | 04. Texas | Fort Hood | 3 | KT883550 | - | - | KT882850 | KT883065 | - | - | - | KT883244 | KT883499 |
| VA141 | Vireo atricapilla | USA | 04. Texas | Fort Hood | 3 | KT883551 | - | - | KT882825 | - | - | KT882771 | KT882888 | - | KT883539 |
| VA142 | Vireo atricapilla | USA | 04. Texas | Fort Hood | 3 | KT883552 | KT883355 | - | KT882840 | KT882980 | KT882690 | - | KT882926 | KT883248 | KT883515 |
| VA143 | Vireo atricapilla | USA | 04. Texas | Fort Hood | 3 | KT883553 | KT883344 | - | KT882827 | KT883026 | KT882691 | KT882739 | KT882886 | KT883278 | KT883441 |
| VA144 | Vireo atricapilla | USA | 04. Texas | Fort Hood | 3 | KT883554 | KT883354 | - | KT882829 | KT883039 | KT882682 | KT882720 | KT882891 | KT883291 | KT883516 |
| VA145 | Vireo atricapilla | USA | 04. Texas | Fort Hood | 3 | KT883555 | KT883308 | - | KT882813 | KT883004 | KT882664 | KT882780 | KT882920 | KT883294 | KT883442 |
| VA114 | Vireo atricapilla | USA | 05. Texas | Balcones Canyon Lands | 2 | FJ560684 | KT883351 | - | KT882841 | KT883053 | KT882648 | KT882727 | KT882912 | KT883211 | - |
| VA156 | Vireo atricapilla | USA | 05. Texas | Balcones Canyon Lands | 3 | - | KT883347 | - | - | KT883051 | KT882631 | - | - | KT883210 | KT883490 |
| VA162 | Vireo atricapilla | USA | 05. Texas | Balcones Canyon Lands | 3 | - | KT883362 | KT883382 | KT882802 | KT883007 | KT882704 | KT882742 | KT882950 | KT883253 | KT883533 |
| VA164 | Vireo atricapilla | USA | 05. Texas | Balcones Canyon Lands | 3 | - | KT883323 | KT883436 | KT882828 | KT883005 | KT882635 | KT882721 | KT882940 | KT883277 | KT883489 |
| VA175 | Vireo atricapilla | USA | 06. Texas | San Antonio | 1 | - | - | KT883426 | KT882799 | KT883076 | KT882629 | KT882773 | KT882966 | KT883220 | KT883495 |
| VA176 | Vireo atricapilla | USA | 06. Texas | San Antonio | 1 | - | KT883314 | KT883427 | KT882817 | KT882984 | KT882667 | KT882767 | KT882895 | KT883221 | KT883519 |
| VA177 | Vireo atricapilla | USA | 06. Texas | San Antonio | 1 | - | - | - | KT882843 | KT883017 | KT882657 | KT882759 | KT882903 | KT883263 | KT883523 |
| VA178 | Vireo atricapilla | USA | 06. Texas | San Antonio | 1 | - | KT883369 | KT883398 | KT882814 | KT883072 | KT882636 | - | KT882951 | KT883252 | KT883463 |
| VA179 | Vireo atricapilla | USA | 06. Texas | San Antonio | 1 | - | KT883300 | KT883409 | KT882872 | KT883046 | KT882675 | KT882793 | KT882972 | KT883231 | KT883534 |
| VA180 | Vireo atricapilla | USA | 06. Texas | San Antonio | 1 | - | KT883317 | KT883423 | KT882871 | KT883073 | KT882637 | KT882781 | KT882900 | KT883259 | KT883454 |
| VA181 | Vireo atricapilla | USA | 06. Texas | San Antonio | 1 | - | KT883364 | KT883370 | KT882811 | KT883018 | KT882672 | KT882765 | KT882916 | KT883193 | - |
| VA182 | Vireo atricapilla | USA | 06. Texas | San Antonio | 1 | - | - | KT883418 | KT882873 | KT882986 | KT882639 | KT882756 | KT882901 | KT883223 | KT883460 |
| VA183 | Vireo atricapilla | USA | 06. Texas | San Antonio | 1 | - | - | - | KT882835 | - | KT882684 | - | - | - | KT883532 |
| VA185 | Vireo atricapilla | USA | 06. Texas | San Antonio | 1 | - | KT883301 | - | KT882818 | KT883043 | KT882644 | KT882732 | KT882962 | KT883191 | KT883524 |
| VA115 | Vireo atricapilla | USA | 07. Texas | Kerr Wildlife Management A. | 2 | FJ560685 | - | KT883384 | KT882795 | KT883054 | KT882649 | KT882760 | KT882890 | KT883247 | KT883507 |
| VA116 | Vireo atricapilla | USA | 07. Texas | Kerr Wildlife Management A. | 2 | FJ560686 | KT883342 | KT883417 | KT882847 | KT883060 | KT882650 | KT882751 | KT882892 | KT883202 | KT883504 |
| VA117 | Vireo atricapilla | USA | 07. Texas | Kerr Wildlife Management A. | 2 | FJ560687 | KT883319 | KT883385 | KT882838 | KT883067 | KT882711 | KT882745 | KT882883 | KT883240 | KT883514 |
| VA149 | Vireo atricapilla | USA | 07. Texas | Kerr Wildlife Management A. | 3 | - | KT883346 | KT883415 | KT882839 | - | KT882641 | KT882743 | KT882878 | KT883276 | KT883500 |
| VA150 | Vireo atricapilla | USA | 07. Texas | Kerr Wildlife Management A. | 3 | - | KT883363 | - | - | KT883041 | - | - | - | KT883255 | KT883450 |
| VA151 | Vireo atricapilla | USA | 07. Texas | Kerr Wildlife Management A. | 3 | - | KT883329 | - | - | KT882982 | - | KT882736 | KT882919 | KT883265 | KT883521 |
| VA152 | Vireo atricapilla | USA | 07. Texas | Kerr Wildlife Management A. | 3 | - | KT883297 | KT883414 | KT882801 | KT883042 | KT882703 | KT882792 | KT882936 | KT883243 | KT883451 |
| VA153 | Vireo atricapilla | USA | 07. Texas | Kerr Wildlife Management A. | 3 | - | KT883343 | - | - | KT883008 | KT882643 | KT882755 | KT882973 | KT883292 | KT883502 |
| VA199 | Vireo atricapilla | USA | 07. Texas | Kerr Wildlife Management A. | 1 | - | - | KT883381 | KT882797 | KT883047 | KT882669 | KT882762 | KT882898 | KT883260 | KT883485 |
| VA200 | Vireo atricapilla | USA | 07. Texas | Kerr Wildlife Management A. | 1 | - | - | KT883416 | KT882875 | KT883055 | KT882681 | KT882750 | KT882914 | KT883228 | KT883478 |
| VA201 | Vireo atricapilla | USA | 07. Texas | Kerr Wildlife Management A. | 1 | - | KT883331 | KT883391 | KT882842 | KT883070 | KT882671 | KT882744 | KT882882 | KT883226 | KT883476 |
| VA202 | Vireo atricapilla | USA | 07. Texas | Kerr Wildlife Management A. | 1 | - | KT883352 | KT883378 | KT882810 | KT883016 | KT882686 | KT882788 | KT882974 | KT883190 | KT883484 |
| VA203 | Vireo atricapilla | USA | 07. Texas | Kerr Wildlife Management A. | 1 | - | - | KT883392 | KT882816 | KT883022 | KT882695 | KT882724 | KT882978 | KT883288 | KT883465 |
| VA204 | Vireo atricapilla | USA | 07. Texas | Kerr Wildlife Management A. | 1 | - | KT883337 | KT883386 | KT882857 | KT882999 | KT882688 | KT882766 | KT882968 | KT883227 | KT883461 |
| VA205 | Vireo atricapilla | USA | 07. Texas | Kerr Wildlife Management A. | 1 | - | KT883310 | KT883419 | KT882874 | KT882988 | KT882696 | KT882794 | KT882959 | KT883192 | KT883493 |
| VA206 | Vireo atricapilla | USA | 07. Texas | Kerr Wildlife Management A. | 1 | - | - | KT883422 | KT882855 | KT882979 | KT882632 | KT882753 | KT882908 | KT883194 | KT883474 |
| VA207 | Vireo atricapilla | USA | 07. Texas | Kerr Wildlife Management A. | 1 | - | - | KT883411 | KT882845 | KT882996 | KT882645 | - | KT882909 | KT883285 | KT883475 |
| VA208 | Vireo atricapilla | USA | 07. Texas | Kerr Wildlife Management A. | 1 | - | KT883306 | KT883428 | KT882812 | KT883045 | KT882677 | KT882722 | KT882907 | KT883225 | KT883494 |
| VA053 | Vireo atricapilla | USA | 08. Texas | Dobbs Mountains | 2 | FJ560649 | KT883321 | - | - | KT883006 | - | - | KT882945 | KT883197 | KT883535 |
| VA054 | Vireo atricapilla | USA | 08. Texas | Dobbs Mountains | 2 | FJ560650 | KT883309 | KT883375 | - | KT883075 | - | KT882770 | KT882939 | KT883274 | KT883447 |
| VA056 | Vireo atricapilla | USA | 08. Texas | Dobbs Mountains | 2 | FJ560652 | KT883326 | KT883374 | KT882823 | KT882997 | KT882705 | KT882785 | KT882955 | KT883236 | KT883522 |
| VA044 | Vireo atricapilla | USA | 09. Texas | Kickapoo Caverns | 2 | FJ560641 | KT883349 | KT883405 | - | KT883009 | KT882707 | KT882738 | KT882928 | KT883199 | KT883525 |
| VA045 | Vireo atricapilla | USA | 09. Texas | Kickapoo Caverns | 2 | FJ560642 | - | - | - | KT883011 | - | - | KT882931 | KT883233 | KT883503 |
| VA046 | Vireo atricapilla | USA | 09. Texas | Kickapoo Caverns | 2 | FJ560643 | - | - | - | KT883034 | - | - | KT882930 | KT883203 | KT883543 |
| VA047 | Vireo atricapilla | USA | 09. Texas | Kickapoo Caverns | 2 | FJ560644 | - | - | - | - | - | - | - | KT883205 | - |
| VA048 | Vireo atricapilla | USA | 09. Texas | Kickapoo Caverns | 2 | FJ560645 | KT883327 | - | - | KT883010 | - | - | - | KT883235 | KT883501 |
| VA049 | Vireo atricapilla | USA | 09. Texas | Kickapoo Caverns | 2 | FJ560646 | KT883318 | - | - | KT883069 | KT882710 | - | KT882929 | KT883245 | KT883446 |
| VA050 | Vireo atricapilla | USA | 09. Texas | Kickapoo Caverns | 2 | - | - | - | - | KT882992 | - | - | KT882934 | KT883181 | KT883452 |
| VA051 | Vireo atricapilla | USA | 09. Texas | Kickapoo Caverns | 2 | FJ560647 | KT883302 | - | - | - | - | - | KT882932 | KT883234 | KT883527 |
| VA186 | Vireo atricapilla | USA | 09. Texas | Kickapoo Caverns | 1 | - | KT883307 | KT883408 | KT882819 | KT883020 | KT882642 | KT882783 | KT882915 | KT883251 | KT883462 |
| VA187 | Vireo atricapilla | USA | 09. Texas | Kickapoo Caverns | 1 | - | - | - | KT882869 | KT883021 | - | - | KT882899 | KT883229 | KT883459 |
| VA188 | Vireo atricapilla | USA | 09. Texas | Kickapoo Caverns | 1 | - | - | - | - | - | - | - | - | KT883295 | - |
| VA189 | Vireo atricapilla | USA | 09. Texas | Kickapoo Caverns | 1 | - | - | KT883400 | KT882809 | KT883030 | KT882656 | KT882791 | KT882952 | KT883264 | KT883496 |
| VA190 | Vireo atricapilla | USA | 09. Texas | Kickapoo Caverns | 1 | - | - | KT883397 | - | - | KT882685 | KT882764 | KT882969 | KT883254 | KT883544 |
| VA191 | Vireo atricapilla | USA | 09. Texas | Kickapoo Caverns | 1 | - | - | - | KT882808 | - | - | - | - | - | - |
| VA192 | Vireo atricapilla | USA | 09. Texas | Kickapoo Caverns | 1 | - | - | KT883396 | KT882863 | KT882981 | KT882676 | KT882728 | KT882904 | KT883195 | KT883477 |
| VA193 | Vireo atricapilla | USA | 09. Texas | Kickapoo Caverns | 1 | - | - | KT883379 | KT882807 | KT882987 | KT882680 | KT882769 | KT882905 | KT883258 | KT883453 |
| VA147 | Vireo atricapilla | USA | 10. Texas | Devil's Ridge | 3 | - | KT883361 | KT883390 | - | - | - | KT882786 | KT882941 | KT883239 | KT883542 |
| VA148 | Vireo atricapilla | USA | 10. Texas | Devil's Ridge | 3 | - | - | - | - | KT883013 | - | - | - | - | - |
| VA194 | Vireo atricapilla | USA | 10. Texas | Devil's Ridge | 1 | - | KT883330 | - | KT882796 | KT883029 | KT882630 | - | KT882897 | KT883289 | KT883538 |
| VA195 | Vireo atricapilla | USA | 10. Texas | Devil's Ridge | 1 | - | KT883357 | KT883433 | KT882859 | KT883058 | KT882679 | - | KT882880 | KT883256 | KT883497 |
| VA196 | Vireo atricapilla | USA | 10. Texas | Devil's Ridge | 1 | - | KT883341 | KT883425 | KT882862 | KT882998 | KT882678 | KT882763 | KT882881 | KT883257 | KT883492 |
| VA197 | Vireo atricapilla | USA | 10. Texas | Devil's Ridge | 1 | - | KT883338 | KT883401 | KT882858 | KT883036 | KT882698 | KT882775 | KT882977 | KT883217 | KT883464 |
| VA069 | Vireo atricapilla | USA | 11. Texas | Independence Creek | 2 | FJ560662 | - | - | - | KT883049 | - | - | KT882947 | KT883286 | KT883529 |
| VA165 | Vireo atricapilla | USA | 11. Texas | Independence Creek | 1 | - | KT883313 | KT883387 | - | KT883044 | KT882658 | KT882782 | - | KT883216 | KT883466 |
| VA166 | Vireo atricapilla | USA | 11. Texas | Independence Creek | 1 | - | KT883311 | KT883393 | KT882846 | KT883024 | KT882661 | KT882749 | KT882961 | KT883188 | KT883530 |
| VA167 | Vireo atricapilla | USA | 11. Texas | Independence Creek | 1 | - | KT883315 | KT883413 | KT882844 | KT883027 | KT882673 | KT882768 | KT882958 | KT883187 | KT883471 |
| VA168 | Vireo atricapilla | USA | 11. Texas | Independence Creek | 1 | - | KT883334 | KT883410 | KT882837 | KT883019 | KT882662 | - | KT882967 | KT883281 | KT883545 |
| VA169 | Vireo atricapilla | USA | 11. Texas | Independence Creek | 1 | - | KT883312 | KT883438 | KT882870 | KT883057 | KT882665 | KT882774 | KT882976 | KT883222 | KT883472 |
| VA170 | Vireo atricapilla | USA | 11. Texas | Independence Creek | 1 | - | KT883333 | KT883424 | KT882815 | KT883035 | KT882666 | KT882790 | KT882971 | KT883200 | KT883470 |
| VA171 | Vireo atricapilla | USA | 11. Texas | Independence Creek | 1 | - | KT883316 | KT883403 | KT882860 | KT882983 | KT882670 | KT882714 | KT882960 | KT883219 | KT883473 |
| VA172 | Vireo atricapilla | USA | 11. Texas | Independence Creek | 1 | - | KT883360 | KT883395 | KT882856 | KT883056 | KT882663 | KT882761 | KT882902 | KT883189 | KT883479 |
| VA173 | Vireo atricapilla | USA | 11. Texas | Independence Creek | 1 | - | KT883335 | KT883394 | KT882861 | KT883071 | KT882633 | KT882772 | KT882896 | KT883198 | KT883455 |
| VA174 | Vireo atricapilla | USA | 11. Texas | Independence Creek | 1 | - | KT883332 | KT883399 | KT882868 | KT883066 | KT882668 | KT882748 | KT882970 | KT883224 | KT883486 |
| VA094 | Vireo atricapilla | Mexico | 12. Coahuila | Pájaros Azules | 2 | FJ560668 | - | - | - | - | - | KT882737 | - | KT883218 | - |
| VA090 | Vireo atricapilla | Mexico | 12. Nuevo León | Rancho Minas Viejas | 2 | FJ560664 | - | - | - | - | - | - | KT882944 | KT883249 | KT883481 |
| VA091 | Vireo atricapilla | Mexico | 12. Nuevo León | Rancho Minas Viejas | 2 | FJ560665 | - | - | - | - | - | - | - | KT883250 | - |
| VA092 | Vireo atricapilla | Mexico | 12. Nuevo León | Rancho Minas Viejas | 2 | FJ560666 | - | - | - | KT883068 | - | KT882740 | KT882943 | KT883287 | - |
| VA093 | Vireo atricapilla | Mexico | 12. Tamaulipas | Carretera a Miquihuana km 9-11 | 2 | FJ560667 | - | - | - | - | - | - | KT882949 | KT883242 | - |
| VA095 | Vireo atricapilla | Mexico | 12. Tamaulipas | Carretera a Miquihuana km 9-11 | 2 | FJ560669 | - | - | - | KT882995 | - | KT882725 | KT882946 | KT883177 | KT883456 |
| VA096 | Vireo atricapilla | Mexico | 12. Tamaulipas | Carretera a Miquihuana km 9-12 | 2 | FJ560670 | - | - | - | KT883052 | - | KT882713 | KT882938 | KT883275 | KT883482 |
| VA097 | Vireo atricapilla | Mexico | 12. Tamaulipas | Carretera a Miquihuana km 9-13 | 2 | FJ560671 | - | - | - | KT883061 | - | - | KT882948 | - | KT883480 |
| VA099 | Vireo atricapilla | Mexico | 12. Tamaulipas | Carretera a Miquihuana km 9-14 | 2 | FJ560673 | - | - | - | - | - | KT882754 | KT882937 | KT883196 | - |
| VA100 | Vireo atricapilla | Mexico | 12. Tamaulipas | Carretera a Miquihuana km 9-15 | 2 | FJ560674 | - | - | - | KT883062 | - | KT882757 | KT882877 | KT883204 | - |
| VA103 | Vireo atricapilla | Mexico | 12. Tamaulipas | Carretera a Miquihuana km 9-16 | 2 | - | - | - | KT882852 | KT883077 | KT882694 | KT882723 | KT882965 | KT883209 | KT883511 |
|  |  |  |  |  |  |  |  |  |  |  |  |  |  |  |  |
|  |  |  |  |  |  |  |  |  |  |  |  |  |  |  |  |
|  |  |  |  |  |  |  |  |  |  |  |  |  |  |  |  |
|  |  |  |  |  |  |  |  |  |  |  |  |  |  |  |  |
|  |  |  |  |  |  |  |  |  |  |  |  |  |  |  |  |
|  |  |  |  |  |  |  |  |  |  |  |  |  |  |  |  |
|  |  |  |  |  |  |  |  |  |  |  |  |  |  |  |  |
|  |  |  |  |  |  |  |  |  |  |  |  |  |  |  |  |
|  |  |  |  |  |  |  |  |  |  |  |  |  |  |  |  |
|  |  |  |  |  |  |  |  |  |  |  |  |  |  |  |  |
|  |  |  |  |  |  |  |  |  |  |  |  |  |  |  |  |

**Supplementary Table 2**. **Primers used for DNA amplification and sequencing.** Part (A) includes all the new primers designed for this study to target fragmented DNA from feathers. Sequences are given in a 5’ – 3’ direction. Melting temperature (Tm) is given in degrees Celsius. (See Materials and Methods for PCR conditions.) Product length is given in base-pairs. Part (B) includes primers from the literature used on samples with high quality DNA.

| (A) |  |  |  |  |  |  |
| --- | --- | --- | --- | --- | --- | --- |
| Forward | Sequence | Tm (basic) | Reverse | Sequence | Tm (basic) | Product Length |
| MC1R_Vaf | CGTGTCTTCCCTCTCCTTCCT | 56.3 | MC1R_VAr2 | TTCTGCTGGCTGGAGATGCTGT | 56.7 | 294 |
| ADAMS6_VAf3 | GGTCAGTTGTTAAGCCTTCTGA | 53 | ADAMS6_VAr4 | GGAATATTCCTCATGTATCATCTC | 52.3 | 291 |
| AETC-F | TGGACTTCAAATCCCCCGATGATCCCAGC | 64.3 | AETC_R2 | GAGTGGTACCAAAGTGCTGCAGACAAGTCC | 64.4 | 278 |
| FIB5_Vaf | GCATATTAATTTATCGATGCTACCAA | 51.7 | FIB5_VAr2 | AAATGAGTCAGGCTGTCCTTAT | 51.1 | 256 |
| GAPDH-F | ACCTTTCATGCGGGTGCTGGCATTGC | 62.7 | GAPDH_Var | CCTCTGAGGGGAAAGGAGGCAGC | 62.4 | 236 |
| TGFB2_Vaf | GGGARGTGTCATGCAGTTGTT | 52.4-54.4 | TGFB2.I5_VAr2 | GTGAAGGTGATAATTTGGCTCTAAC | 54.4 | 235 |
| TROP.6_Vaf | ACCTCCTGTGCAGCCAAAAGA | 54.4 | TROP.6_VAr | GAAAGGGGCGGATGGAAGATT | 54.4 | 280 |
| IQGAP2_VAF | GTGCTGAGCTAGGACTCTC | 53.2 | IQGAP2_VAR | TGCCCACTCCCTTGAAACTC | 53.8 | 249 |
| LAMA2_F | CCAAGAAGCAGCTGCAGGATGAGATGC | 53.2 | LAMA2_R | CTGCCGCCCGTTGTCGATCTCCACCAG | 53.8 | 295 |
|  |  |  |  |  |  |  |
| (B) |  |  |  |  |  |  |
| Locus | Primer pairs | Reference |  |  |  |  |
| ND2 | L5215 and H1064 | Zink et al. (2010) Auk 127: 797-806 | |  |  |  |
| MCIR | MSH8 and MSH9 | MacDougall-Shackleton et al. (2003) Mol. Biol. Evol. 20: 1675-1681 | | |  |  |
| ADAMS6 | ADAMS6-F and ADAMS6-R | Backström et al. (2006) Genetics 174: 377-386 | | |  |  |
| AETC | AETC-F and AETC-R | Primmer et al. (2002) Mol. Ecol. 11: 603-612 | | |  |  |
| FIB5 | Fib5 and Fib6 | Kimball et al. (2008) Mol. Phylogen. Evol. 50: 654-660 | | |  |  |
| GAPDH | GAPDH-F and GAPDH-R | Borge et al. (2005) Genetics 171: 1861-1873 | | |  |  |
| TGFB2I5 | TGFB2-F and TGFB2-R | Borge et al. (2005) Genetics 171: 1861-1873 | | |  |  |
| TROP6 | Trop.6F and Trop.6R | Kimball et al. (2008) Mol. Phylogen. Evol. 50: 654-660 | | |  |  |
| IQGAP2 | IQGAP2-F and IQGAP2-F | Backström et al. (2006) Genetics 174: 377-386 | | |  |  |
| LAMA2 | Same as in Part (A) | Borge et al. (2005) Genetics 171: 1861-1873 | | |  |  |
|  |  |  |  |  |  |  |

**Supplementary Figure 1**. **Comparisons of Extended Bayesian Skyline Plot demographies.** Plots include mitochondrial DNA (ND2), sex-linked (Z chromosome) loci, and all loci (Figure 5). All effective population sizes converged to similar median estimates but in single or dual loci EBSPs credibility intervals were too wide to reject a constant population size (*i.e.* zero population growth events). Population size values (y-axis) are given in log_10_ units.
